# Ice as a Green-Structure-Directing Agent in the Synthesis of Macroporous MWCNTs and Chondroitin Sulphate Composites

**DOI:** 10.3390/ma10040355

**Published:** 2017-03-28

**Authors:** Stefania Nardecchia, María Concepción Serrano, Sara García-Argüelles, Marcelo E. H. Maia Da Costa, María Luisa Ferrer, María C. Gutiérrez

**Affiliations:** 1 Materials Science Factory, Instituto de Ciencia de Materiales de Madrid (ICMM), Consejo Superior de Investigaciones Científicas (CSIC), Campus de Cantoblanco, C/Sor Juana Inés de la Cruz 3, 28049-Madrid, Spain; sara_g.arguelles@yahoo.es (S.G.-A.); mferrer@icmm.csic.es (M.L.F.); mcgutierrez@icmm.csic.es (M.C.G.); 2 Departamento de Física, Pontificia Universidade Católica do Rio de Janeiro (PUC-Rio), Rua Marquês de São Vicente 225, Gavea 22451-900, Rio de Janeiro, Brazil; maiacosta@vdg.fis.puc-rio.br; 3 Laboratory of Interfaces for Neural Repair, Hospital Nacional de Parapléjicos, Servicio de Salud de Castilla-La Mancha, Finca de la Peraleda s/n, 45071-Toledo, Spain; mslopezterradas@sescam.jccm.es; 4 Departamento de Tecnología Química y Energética, Tecnologia Química y Ambiental y Tecnología Mecánica y Química Analítica, Universidad Rey Juan Carlos, 28933-Madrid, Spain

**Keywords:** chondroitin sulphate, freeze-casting, hierarchical materials, multi-walled carbon nanotubes, porous materials, scaffold

## Abstract

The incorporation of multi-walled carbon nanotubes (MWCNTs) into chondroitin sulphate-based scaffolds and the effect on the structural, mechanical, conductive, and thermal properties of the resulting scaffolds is investigated. Three-dimensional hierarchical materials are prepared upon the application of the ice segregation-induced self-assembly (ISISA) process. The use of ice as structure-directing agents avoids chemicals typically used for this purpose (e.g., surfactants, block copolymers, etc.), hence, emphasising the green features of this soft-templating approach. We determine the critical parameters that control the morphology of the scaffolds formed upon ice-templating (i.e., MWCNTs type, freezing conditions, polymer and MWCNT concentration). MWCNTs are surface functionalized by acidic treatment. MWCNT functionalization is characterized by Raman, Fourier transfer infrared (FTIR) and X-ray Photoelectron (XPS) spectroscopies. Scanning electron microscopy (SEM) analysis and porosity studies reveal that MWCNT content modifies the morphology of the macroporous structure, which decreases by increasing MWCNT concentration. Differences in scaffold morphology should be translated into their conductivity and mechanical properties. As a general trend, the Young’s modulus and the electrical conductivity of the scaffolds increase with the MWCNT content. Preliminary biocompatibility tests with human osteoblast-like cells also reveal the capability of these structures to support cell growth.

## 1. Introduction

Since pioneering work by Iijima in 1991 [1], carbon nanotubes (CNTs) have been the subject of numerous studies given their unique properties, including extremely high electrical and thermal conductivities, outstanding mechanical properties, and ability to form interconnected porous networks with high surface areas.

A significant amount of research has been performed on the assembly of CNTs into three-dimensional (3D) macrostructures [2,3]. The incorporation of CNTs into polymer-based composites has arisen as an attractive strategy frequently pursued, but the fabrication of 3D architectures, with the use of natural polymers, has been more rarely accomplished [4]. To date, several approaches have been developed to achieve this goal. For instance, processes based on unidirectional freezing and subsequent freeze-drying, such as the ice segregation-induced self-assembly (ISISA) technique [5], are gaining increased interest since they have been proven as excellent techniques for creating scaffolds with sophisticated, hierarchical, and interconnected porous 3D architectures either with or without CNTs [6,7]. Ice-templating involves the use of ice crystals as a template for building up hierarchical 3D structures, with the ice crystals being formed by freezing an aqueous suspension [8]. This methodology, which meets the standards of green chemistry, is highly biocompatible as it relies on the use of aqueous suspensions or hydrogels and runs in the absence of further chemical reactions [9,10]. More importantly, the properties of the scaffolds, such as structure [11], crystallinity [12], mechanical strength [13], and conductivity [14], can be modulated by changing the freeze-drying conditions and the composition of the solution. During ice-templating, the pore structure is significantly affected by the growth of the ice crystals, which at the same time is governed by the initial composition of the suspension as well as by the freezing process itself [7,15,16,17]. This is why the ISISA technique is considered as a simple and versatile bottom-up process for the fabrication of 3D macrostructures with controlled pore sizes and structure. In our group, we have previously described the application of the ISISA process to aqueous dispersions of CNTs. Particularly, we applied this technique to a dispersion of HNO_3_-treated multi-walled CNTs (MWCNTs) in an aqueous solution of chitosan for the fabrication of MWCNT-based 3D scaffolds useful in bone tissue regeneration [10]. When homogeneously mineralized by a “flow-through” electrodeposition process, these scaffolds were able to enhance osteoblast terminal differentiation [18]. Additionally, this methodology allowed the successful preparation of biocompatible CNT-based scaffolds with other biopolymers, such as chondroitin sulphate (CS) and gelatin, thus allowing the achievement of 3D substrates with different hierarchical and morphological features at the microscale to further modulate cell responses [15].

Herein, we report on some of the parameters that are critical for the control the morphology of 3D CNT-based macrostructures. These matrices were obtained by the application of the ISISA process into aqueous suspensions containing MWCNTs and CS, which acted as a dispersant agent [19]. CS belongs to the glycosaminoglycan family. It is an alternating copolymer of β-(1,4)-d-glucuronic acid and β-(1,3)-*N*-acetyl-d-galactosamine that is sulphated either at the 4-position or at the 6-position [20]. Inclusion of CS in the scaffold structure may promote the secretion of proteoglycan and type II collagen, and influence the bioactivity of the seeded chondrocytes [21,22], thus influencing cellular responses. Furthermore, the scaffold structure was modified by the incorporation of two different types of functionalized MWCNTs (i.e., long MWCNTs and short MWCNTs; abbreviated as LN and SN, respectively) [15]. In order to thoroughly control the assembling of CNTs into 3D macrostructures, we systematically investigated the effect of polymer and MWCNT concentration and freezing conditions (immersion rate) on the properties of the resulting structures. The morphology and topography of the so-prepared scaffolds were characterized by scanning electron microscopy (SEM). Additionally, the mechanical, electrical and thermal properties of the scaffolds were assessed by dynamic mechanic analyser, the four-point method and thermogravimetry analysis, respectively. Water adsorption and porosity of these 3D structures were also determined. Finally, preliminary biocompatibility tests in vitro, including viability and morphology, were performed with human osteoblast-like Saos-2 cells to identify any potential toxic effects of these scaffolds on mammalian cells.

## 2. Results and Discussion

### 2.1. Characterization of Modified MWCNTs

The scaffold morphology was modified by the incorporation of two different types of MWCNTs, LN and SN. The former (e.g., LN) were obtained upon acid treatment with HNO_3_ that resulted in MWCNT functionalization with oxygenated groups without significant modification of their original length (i.e., 5–9 µm) (Figure S1a,b). An oxidative acid treatment is often used for nanotube purification. As a result of the oxidizing acid attack, the ends and surfaces of the nanotubes become covered with oxygen-containing groups, such as carboxylate and ether groups. HNO_3_ is the most common reagent for the oxidation of nanotubes, which can selectively remove amorphous carbon and metal catalysts and, at the same time, generate oxygenated groups at the exposed CNT surface [23]. However, acid attack, most frequently performed under boiling conditions, is accompanied by severe degradation effects, including material’s loss and selective removal of metallic nanotubes, tube shortening, as well as the formation of structural defects and carbonaceous debris [24,25,26]. The latter (e.g., SN) were obtained upon the application of a more aggressive acid treatment with a mixture of HNO_3_ and H_2_SO_4_ that not only promoted the functionalization with oxygenated groups, but also shortened their original length to ca. 3 µm (Figure S1c). Liu et al. used a mixture of concentrated H_2_SO_4_/HNO_3_ (3:1 by volume) to cut highly-tangled long ropes of SWCNTs into short, open-ended pipes and, thus, produced many carboxylic acid groups at the open ends [27].

**Figure 1:**
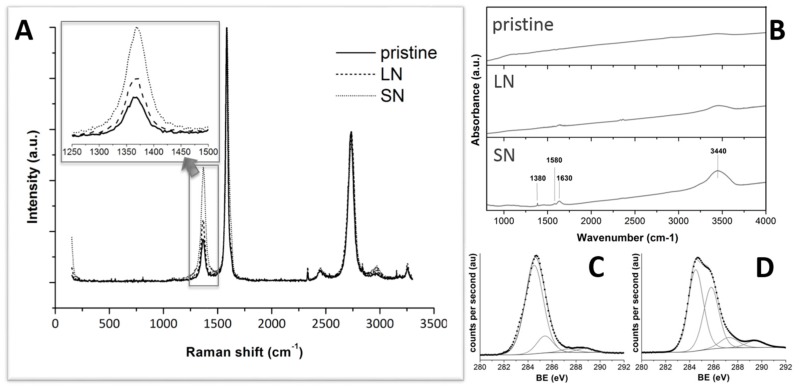
Representative Raman (**A**) and FTIR (**B**) spectra of pristine and modified MWCNTs (LN and SN). XPS C1s spectra of LN (**C**) and SN (**D**) MWCNTs.

Raman, Fourier transfer infrared (FT-IR), and X-ray photoelectron (XPS) spectroscopy measurements were used to investigate the structural changes of MWCNTs caused by the acid treatment. The Raman spectrum of nanotubes is resonantly enhanced if the excitation energy is close to the band-gap transition energies. Thus, Raman spectroscopy can be used to study structural changes in MWCNTs that lead to changes in the electronic structure. In particular, the Raman spectrum of MWCNTs consists of three prominent vibrational groups, which are called the tangential mode or G-band (1580 cm^−1^), the disordered (D) band (1300 cm^−1^), and the binary disordered (2D) band (2600 cm^−1^). The intensity of the D-band is commonly interpreted in terms of the concentration of sp^3^ defects that are present in the nanotubes sidewalls [28]. The two bands at around 1580 and 1350 cm^−1^ in the spectra are assigned to E_2g_ and A_1g_ modes of the graphite sheet, respectively [25,29]. Figure 1A shows the Raman spectrum of the acid-treated MWCNTs. The intensity ratio between the D-line and the G-line of acid-treated MWCNTs clearly exceeded that of unmodified ones. These results resembled the CNT deterioration under strong oxidation conditions. This also indicates that a C=C bond may be broken to form a C–C bond, generating defects in the MWCNTs.

FT-IR spectroscopy allowed us to identify the evolution of the surface functional groups on the CNTs throughout the chemical oxidation process. The absorption bands attributed to carboxylic acids were observed at various wavenumbers, including ca. 3400 cm^−1^ (OH stretching), 1600 cm^−1^ (C=O stretching), and 1300 cm^−1^ (C–O stretching) [30]. However, the bands around 3400 and 1300 cm^−1^ are difficult to define as carboxylic acids since they can also contain contributions from hydroxyl C–OH groups [31]. The FT-IR spectra of the raw and acid-treated MWCNTs (LN and SN, respectively) are shown in Figure 1B. The peak at 1630 cm^−1^ can be attributed to the carbonyl (C=O) absorption band. The peak at 1580 cm^−1^ can be attributed to the anion carboxylate (–COO^−^) [32,33]. The intensity of this peak increased from pristine MWCNTs—nil, in this case—to LN up to SN samples, correlating the extension of the carboxylic functionalization with the strength of the acidic treatment. Nonetheless, carboxylic acid quantification was not possible by using FTIR since the intensity of the band was very small and the band could contain contributions from various functional groups simultaneously [32].

To elucidate the changes in surface states of MWCNTs after modification, XPS analyses were applied. In XPS survey spectra, the binding energies for the C1s and O1s peaks were observed at 284.5 and 531.0 eV, respectively, which are consistent with previously reported values [34,35]. Figure 1C,D show the C1s deconvoluted XPS region of LN and SN, respectively. The C1s spectra were resolved into at least four sub-bands that represent carbon in polyaromatic (sp^2^) structures (B.E. = 284.5 ± 0.1 eV), aliphatic (sp^3^) structures (B.E. = 285.6 ± 0.2 eV), carbonyl or quinone groups (B.E. = 287.6 ± 0.1 eV), and carboxyl or ester groups (B.E. = 288.9 ± 0.3 eV). The assignment of the fitted peaks in the case of the acid-treated sample is consistent with those found in the literature [36]. The fitting curve of the O1s spectrum for LN and SN exhibited two main peaks, as shown in Figure S2. The first peak at 531.5 eV (B.E. = 531.5 ± 0.1 eV) corresponded to oxygen atoms in C–O group and the second peak at 533.7 eV (B.E. = 533.7 ± 0.1 eV) originated from oxygen atoms in C=O and O–C=O groups [37,38]. Therefore, XPS results suggested that the acidic treatment induced the appearance of functional groups—including hydroxyl, carbonyl and carbonate—on MWCNTs, the stronger the acid treatment, the larger the amount of functional groups.

### 2.2. Characterization of MWCNTs/CS Scaffolds

#### 2.2.1. Scaffolds Morphology and Porosity

**Figure 2:**
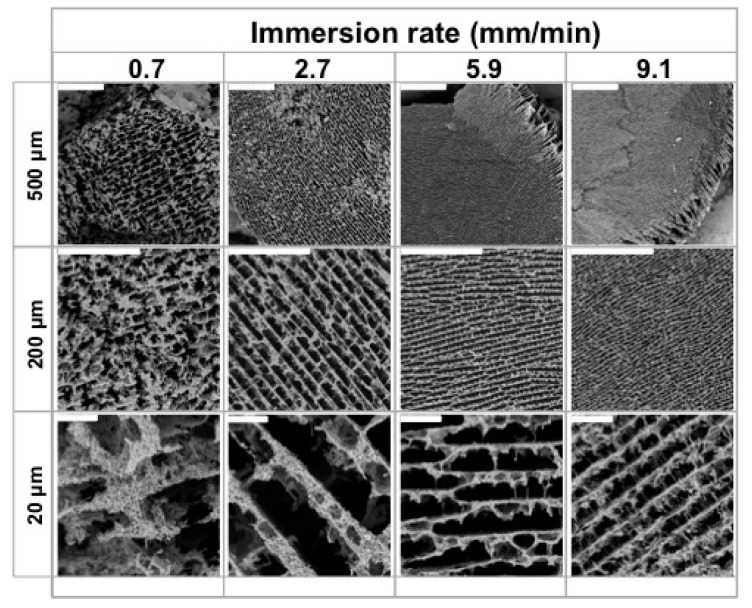
Representative SEM images of cross-sectioned CS1SN6 scaffolds. The dipping rates were 0.7, 2.7, 5.9, and 9.1 mm·min^−1^. Scale bars represent 500, 200, and 20 μm, as indicated in the figure.

As mentioned in the introduction, MWCNT/CS scaffolds were prepared by the ISISA process, which is critical for the achievement of microchanneled-type longitudinal structures. We first studied the use of different immersion rates (e.g., 0.7, 2.7, 5.9, and 9.1 mm·min^−1^) at a fixed MWCNT/CS concentration (CS at 1 wt % and MWCNTs at 6 wt %, named as CS1SN6 or CS1LN6). As in previous works, SEM images revealed that the resulting macrostructure was characterized by micrometer-sized pores well-aligned in the freezing direction for both and SN- and LN-composed MWCNT/CS, since the constant freezing rate determined the distance from the ice front to the liquid nitrogen immersion level and, hence, for the temperature (Figure 2 and Figure S3, respectively) [17,39].

It is worth noting that changes in the pore size of the microchannels also affected the thickness of the matter accumulated between adjacent channels, i.e., the larger the microchannel pore size, the larger the accumulation of matter between channels. Interestingly, scaffolds frozen at 0.7 mm·min^−1^ deviated from this pattern, most likely because the slow advance of the freezing front impeded the formation of well-aligned microchannels.

In the remaining cases, the microstructural features resulting from freezing at different rates are in good agreement with previous results described by Deville et al. [40]. It is stated that slow freezing rates allow the formation of large ice crystals, which are responsible for templating the microchanneled structure. Meanwhile, fast freezing rates favour supercooling and, hence, impede the formation of large ice crystals, so that the microchanneled structure is scaled down. It is worth noting that MWCNT/CS scaffolds prepared at 5.9 mm·min^−1^ (Figure S3) exhibit some slight radial heterogeneity, which is quite noticeable at 9.1 mm·min^−1^, with the outer edge of the scaffold showing a more distinguishable microchanneled structure than the inner part.

**Figure 3:**
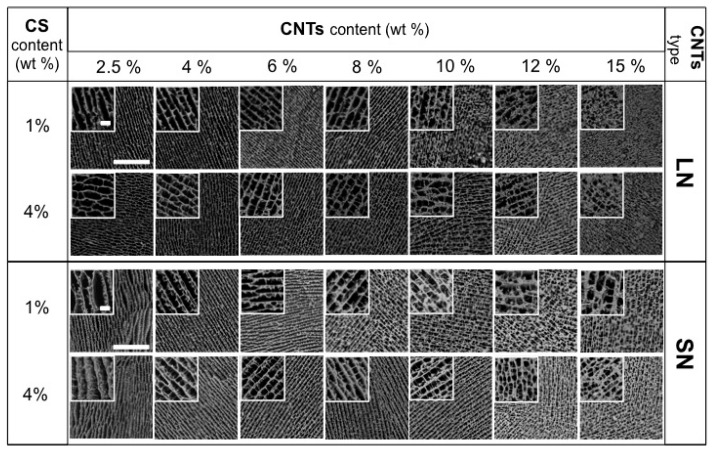
SEM images of cross-sectioned of MWCNT/CS scaffolds prepared with different content of CS and MWCNT (LN or SN) and at a dipping rate of 5.9 mm·min^−1^. Scale bars represent 200 μm and, in inset images, 20 μm.

**Figure 4:**
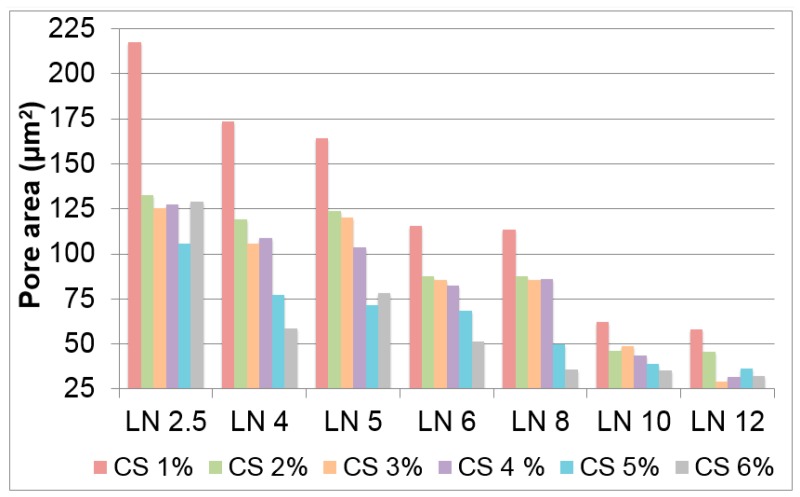
Scaffold porosity of LN-composed MWCNT/CS scaffolds prepared with different contents of CS and MWCNTs.

Radial heterogeneity occurs because of the occurrence of two opposite features. On the one hand, the use of a fast immersion rate further approaches the supercooled regime and even smaller ice crystals (than for dipping rates of 2.7 mm·min^−1^) are formed. On the other hand, and taking into account the low heat capacity and thermal conductivity (latent heat) of liquid nitrogen, there is a limit to the sample mass that nitrogen is capable of supercooling. Above that limit, a nitrogen vapour barrier is formed around the sample (e.g., the Leidenfrost effect), restricting the rate at which heat is withdrawn from the sample and determining the formation of large ice crystals at the outer shell of the monolith. Moreover, the channel size was not only dependent on the immersion rate, but also on the type of CNTs. We then fixed the immersion rate at 5.9 mm·min^−1^ and studied the effect of CS and MWCNT concentration on tailoring scaffold morphology. For low MWCNT contents (ca. 2.5–4 wt %), SEM images (Figure 3) revealed the formation of a layered architecture in the form of poorly interconnected CS sheets arranged in parallel layers. The increase of the MWCNT content (ca. 6 wt %) favoured the formation of pillars crossing between layers, fully interconnecting the 3D structure. Additionally, the size of the porous channels was scaled down, a consequence of the increased difficulty for the ice crystals to form in the presence of CNTs. A further increase of the MWCNT content resulted in structures where the porous channels are almost closed; that is, we hypothesised that the MWCNT content reached values that favour the formation of amorphous (supercooled water) rather than crystalline ice, so that no segregation of matter occurred [41]. SEM images of SN/CS scaffolds revealed similar structural features than those observed for LN/CS scaffolds, demonstrating than CNT concentration had a larger impact on porosity than CNT size. Interestingly, we have also observed the capability of forming macroporous structures upon freezing when the SN content in the aqueous solution is as high as ca. 18 wt % (Figure S4). The above-mentioned results reveal that the MWCNT content indeed modifies the morphology of the CS macrostructure (Figure 4). Moreover, the CS content also played a similar role as the MWCNT content in the resulting structure, with pore sizes that decreased with the CS content (Figure 4).

#### 2.2.2. Thermogravimetric Analysis (TGA)

**Figure 5:**
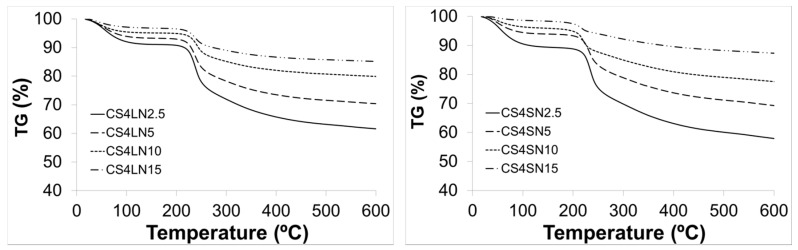
TGA thermograms of MWCNT/CS scaffolds.

The thermal stability of scaffolds was assessed by TGA analysis. Scaffold weight loss in percentage (%) is plotted in relation to temperature treatment (Figure 5). For MWCNT/CS scaffolds, two-stage TG curves were observed. The first one started at around 41 °C and reached a maximum at ca. 92 °C, assigned to the loss of water. The second stage started at around 196 °C and reached a maximum at 252 °C, corresponding to the decomposition (thermal and oxidative) of CS and vaporization and elimination of volatile products. In this graph, CS weight loss was observed at ca. 229 °C which corresponds to CS moieties (Figure S5) [42]. The weight losses agreed with the CS contents of the different scaffolds.

#### 2.2.3. Cross-Linking with Hexamethylene Diisocyanate (HMDI)

As MWCNT/CS scaffolds are highly soluble in water, reinforcement of their architecture was achieved by cross-linking with HMDI. HMDI is a bifunctional molecule with lower cytotoxicity than glutaraldehyde [43]. In general, isocyanates may react with nucleophilic functional groups, such as amines, alcohols, and protonated acids [44]. The formation of cross-linking networks is due to the reaction of isocyanate groups from HMDI with OH/COOH groups from CS and oxidized MWCNTs. When the CS scaffolds were cross-linked with HMDI (Figure S6c,d), the scaffold retained the original structure (Figure S6a,b). MWCNT/CS scaffolds resulting after HMDI treatment (named as CS*x*LN*x*H or CS*x*SN*x*H, being *x* the weight percentage in the scaffold for each component) were quite stable under the experimental conditions used for swelling and cell culture studies. The weight loss of cross-linked scaffolds occurred at three different temperature ranges, as depicted by TG curves (Figure S7). The first two were equivalent to those observed for non-cross-linked ones and the third one started at around 406 °C and reached a maximum at 432 °C. This feature revealed the formation of thermally-stable groups resulting from the cross-linking reaction between CS and HDMI [45]. Further attempts to identify the changes induced by HMDI treatment were done by FTIR spectroscopy. The representative absorption peaks for nascent CS are reported elsewhere [46]: OH and NH stretching (3426 cm^−1^), CH_2_ stretching (2926 cm^−1^), amide I (1634 cm^−1^), COO^−^ *ν*^as^ stretching (1559 cm^−1^), COO^−^ *ν*^s^ stretching (1414 cm^−1^), SO_4_^2−^ related modes (1248 cm^−1^), and C–O stretching (1088 cm^−1^ and 1074 cm^−1^). In the spectrum of the cross-linked CS scaffold (CSH) (Figure S8), we found a new absorption peak at 2283 cm^−1^ which is characteristic of HMDI absorption band and thus indicate the successful cross-linking. One might also wonder whether the OH/COOH groups of the functionalized MWCNT (LN and SN) could also react with HDMI. Unfortunately, FTIR spectra were not conclusive in this regard.

#### 2.2.4. Water-Binding Capacity

The structural stability of the scaffolds was evaluated by SEM studies after swelling (Figure S6e,f). As shown in the micrographs, water uptake did not significantly change the structural morphology of MWCNT/CS scaffolds. Table 1 summarizes the water-binding capacity of these scaffolds. These studies indicate a very high swelling capacity, as the scaffolds were able to retain a high amount of water, much higher than their original weight [47,48]. It is also observed that, as the concentration of CS and/or MWCNTs increased, the swelling ratio decreased. This might be caused by the participation of hydrophilic OH/COOH groups in cross-linking, hence decreasing the overall hydrophilicity of the network. Swelling also increased pore size and total porosity, thus maximizing the internal surface area of the scaffolds, favouring nutrient transport, and facilitating cell infiltration. Furthermore, an increased swelling also allows the samples to avail nutrients from culture media more effectively. On the contrary, swelling could also cause some detrimental effects on the scaffold mechanical properties so, depending on the particular application, swelling extension should be properly tuned for an optimized response.

#### 2.2.5. Conductivity Properties

**Figure 6:**
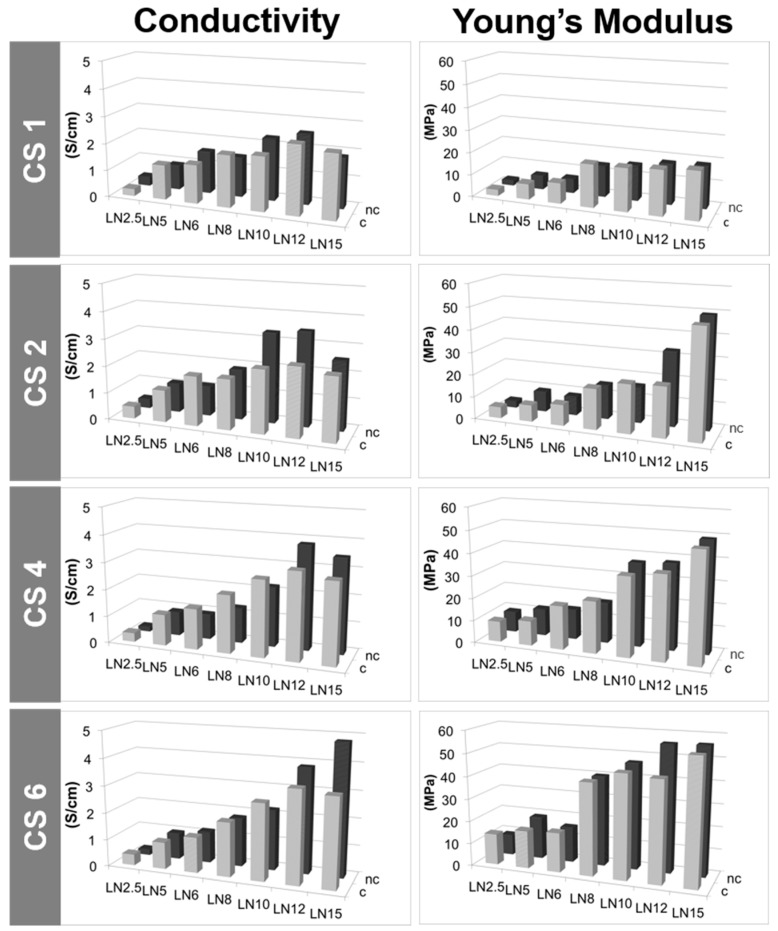
Conductivity and Young’s modulus values of LN-composed MWCNT/CS scaffolds prepared with different concentrations of LN and CS, and either cross-linked (*c*) or non-cross-linked (*nc*).

**Figure 7:**
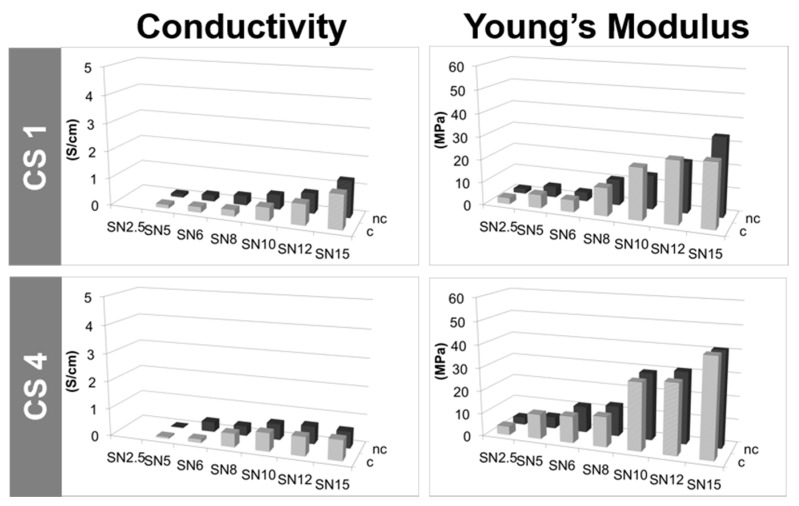
Conductivity and Young’s modulus values of SN-composed MWCNT/CS scaffolds prepared with different concentrations of SN and CS, and either cross-linked (*c*) or non-cross-linked (*nc*).

A key characteristic of MWCNT/CS scaffolds is the electrical conductivity, defined by the charge transfer from one conductive particle to another. The effective electrical conductivity of MWCNT/CS scaffolds depends upon many factors such as size, shape, density, and distribution of MWCNTs, as well as chemical interactions between the two materials composing their structure [49]. In this particular case, the electrical conductivity was most likely dominated by the goodness of the physical contact among the MWCNTs within the scaffold structure. The contact resistance among MWCNTs will depend on their concentration and geometrical arrangement. Thus, differences in morphology (Figure 3) should be translated into the electrical conductive properties of the scaffolds. Figure 6 and Figure 7 show conductivity values for scaffolds prepared with a fixed polymer content (1%, 2%, 4%, and 6% *w*/*w*) and a variable percentage of MWCNTs (from 2.5% to 15% *w*/*w*). It can be observed how electrical conductivity increased with the content of LN, reaching a maximum value of 4.8 Scm^−1^ for CS6LN15 (Figure 6 and Table S1) [16].

The increase of the CS content (1% to 6% *w*/*w*) in scaffolds also having high LN contents (12% to 15% *w*/*w*) resulted in an enhancement of the electrical conductivity (Figure 6 and Table S1) most likely because good LN dispersions at the solution stage result in scaffolds where there are more physical contacts among LNs. By contrast, scaffolds with a lower content of MWCNTs (up to 10% *w*/*w*) did not exhibit significant changes in conductivity by varying the amount of dispersant because of the easier dispersion of MWCNTs in these cases (Figure S9 and Table S1). With regard to HMDI treatment, cross-linking not only favoured the structural stability, but also improved the conductivity at low contents of MWCNTs (up to 8% *w*/*w*). The behaviour of scaffolds prepared with SN content (Figure 7 and Table S2), resembled that found for LN reaching a maximum value of 1.9 Scm^−1^ for CS1SN21 (not shown). In this case, electrical conductivities were significantly lower than those found for LN scaffolds. This different behaviour was attributed to the presence of defects coming from functionalization on the SN surface, the number of which was larger than on LN surface. Actually, the lack of improvement of the electrical conductivity after HMDI-cross-linking corroborated this feature (Figure 7 and Table S2).

#### 2.2.6. Mechanical Properties

Structural differences among scaffolds (Figure 3) had an impact on their respective mechanical properties as indicated by the Young’s modulus found for different MWCNT/CS scaffolds. Figure 6 (Table S1) and Figure 7 (Table S2) show the evolution of the tensile strength as a function of the morphology of the sample. The Young’s modulus increased with the MWCNT (LN or SN) content used for the formation of the scaffold macrostructure [50]. High values of Young’s modulus reflected a certain bulky character enhancement in those samples with thicker walls. Nonetheless, low MWCNT contents (e.g., 2.5–6 wt %) resulted in scaffolds with poor mechanical properties. Meanwhile, neither the use of different CS concentrations (keeping fixed the MWCNT concentration) or different freezing rates provided significant differences in the respective mechanical properties of these samples (Figures S9, S10, and Table S3, respectively).

### 2.3. Biocompatibility Studies In Vitro

**Figure 8:**
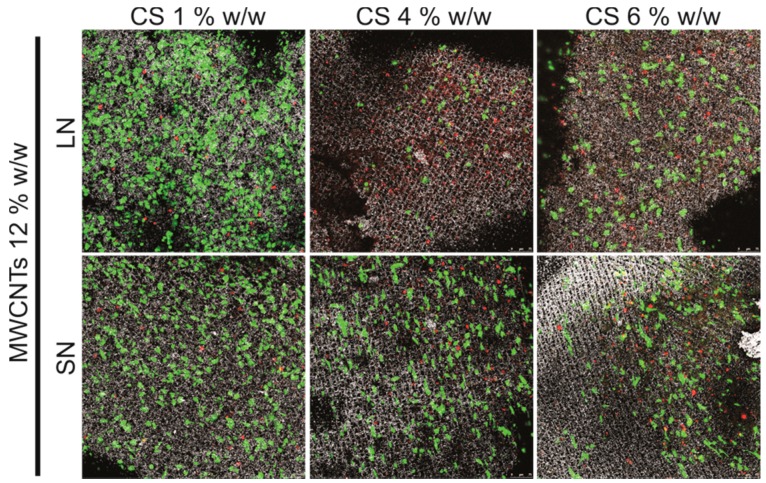
Representative confocal laser scanning microscopy (CLSM) images of Saos-2 cells cultured on MWCNT/CS scaffolds prepared with different CS concentrations and using either LN or SN MWCNTs (12% *w*/*w*). Note that green spots represent live cells while red ones indicate dead ones. Reflexion images from the scaffolds are also merged with fluorescence ones for better visualization of surface topography and relative cell location.

Finally, we carried out preliminary biocompatibility studies in vitro with human osteoblast-like Saos-2 cells to explore the potential use of these 3D porous structures as platforms for guiding cell growth. Figure 8 shows some representative fluorescence images of cells cultured on scaffolds varying the CS content for a fixed MWCNT concentration (12% *w*/*w*). Cell viability (indicated by the ratio of alive cells—in green- with respect to the dead ones—in red-) decreased in scaffolds with higher CS contents (e.g., 4 and 6 wt %). On the contrary, cell viability increased in scaffolds prepared with SN as compared to those prepared with LN, most likely due to already-observed cytocompatibility benefits of short MWCNTs [15]. Micrographs also showed how cells successfully spread on and colonize the scaffolds surface. Cell infiltration within the internal macropores was not observed in any case, most likely hampered by the limited exchange of nutrients and waste products between the outer and the inner parts of the scaffolds.

## 3. Materials and Methods

### 3.1. Materials

For the preparation of the scaffolds, chondroitin 4-sulfate sodium salt (CS, from bovine trachea, LOT: STBB3576), multi-walled carbon nanotubes (MWCNTs, 110–170 nm in diameter, 5–9 μm in length) and hexamethylene diisocyanate (HMDI) were used. Chemical reagents were purchased from Sigma-Aldrich (St. Luis, AZ, USA) and used as received unless otherwise indicated. Cell culture media and supplements were purchased from Lonza (Basel, Switzerland).

### 3.2. Preparation and Characterization of MWCNTs

MWCNTs were first purified and functionalized by acidic treatment with HNO_3_ [51]. Briefly, concentrated HNO_3_ (40 mL, 14 M) were added to MWCNTs (800 mg) in a round-bottom glass flask. The suspension was stirred overnight to guarantee homogenous dispersion of the nanotubes and then heated at 130 °C for 5 h, under a reflux condenser. After cooling at room temperature, it was filtered under vacuum by using cellulose filters with 0.2 μm pore size and several washes with distilled water in order to eliminate any acidic residues from HNO_3_ treatment. Once the pH in the washes was stabilized at 5.5, MWCNTs were freeze-dried for 24 h and stored until used. To simplify, the so-obtained MWCNTs will be named as long MWCNTs (LN). The short MWCNTs (SN) were obtained by simple exposure of LN to a stronger acidic treatment. Particularly, 200 mg of LN were sonicated for 4 h (DT102H, Bandelin Sondez Digitex) in a concentrated solution containing H_2_SO_4_ and HNO_3_ (12 mL, 3:1). The temperature in the bath was maintained below 40 °C along the process. After treatment, the resulting SN were allowed to cool down at room temperature, then repeatedly washed with distilled water and finally freeze-dried as described above. The morphology of the different MWCNTs was investigated with a 200-KeV JEOL JEM-2000FX electron transmission microscope (TEM) (JEOL, Peabody, MA, USA). Raman spectroscopy analysis was performed using a micro-Raman spectrometer (NT-MDT, NTEGRA SPECTRA) with an excitation wavelength of 473 nm equipped with a CCD detector. Fourier transform infrared (FTIR) spectra were recorded between 400 and 4000 cm^−1^ on a Bruker Alpha FT-IR spectrometer. For the X-ray photoelectron spectroscopy (XPS) measurements, we used an Al Kα X-ray source (h*ν* = 1486.58 eV) and an Alpha 110 commercial hemispherical electron energy analyser. XPS spectra were collected at a fixed analyser pass energy of 20 eV and the data were processed using the CasaXPS software (Version 2.3.16 PR1.6, Casa Software Ltd., Teignmouth, UK).

### 3.3. Fabrication of MWCNT/CS Scaffolds

MWCNT/CS scaffolds were prepared by using the ISISA technique as previously described [52]. Briefly, CS solutions were made by dissolving CS in deionized water. Functionalized MWCNTs were then dispersed in the CS solution by stirring at room temperature for 24 h and finally sonicated for 4 min. The MWCNT/CS suspension was then collected into insulin syringes (1 mL) and dipped at a constant rate into a cold bath maintained at a constant temperature of −196 °C (liquid nitrogen). The unidirectionally-frozen samples were then freeze-dried using a thermoSavant Micromodulyo freeze-drier. The resulting monoliths kept both the shape and the size of the insulin syringes (or any other container used to collect the suspensions prior freezing). Diverse series of MWCNT/CS scaffolds were then prepared following this method changing the concentration and type of MWCNTs (LN or SN) and the concentration of CS (Table 1). MWCNT/CS scaffolds were finally exposed to hexamethylene diisocyanate (HMDI) vapours at 37 °C for seven days to achieve cross-linking and structure reinforcement. Cross-linked scaffolds were then aerated for further 24 h. For simplification purposes, scaffolds will be referred in the manuscript as CS*x*LN*x* or CS*x*SN*x*, *x* being the weight percentage of each component. When additional H appears at the end of the name, it stands for HMDI so it refers to cross-linked scaffolds.

### 3.4. Morphological and Physico-Chemical Characteristics of MWCNT/CS Scaffolds

Details of scaffold architecture, cross-section morphology and pore size were examined by using a DSM-950 scanning electron microscope (Zeiss) (Carl Zeiss, Oberkochen, Germany). The inner parts of fractured segments were mounted onto cylindrical aluminum stubs and gold-coated using a sputter coater. Scaffold porosity was measured from SEM images by using the UTHSCSA *ImageTool* software, version 3.00. Briefly, pores were delimited in each scaffold image and their area converted in μm^2^ after calibration with the scale bar. Porosity was then expressed as pore area per μm^2^ (*A_P_*) and pore width (*W_P_*). FTIR measurements were made using Thermo Nicolet-870 FT-IR spectrophotometer in the spectral region 500–4000 cm^−1^, where polymer powders (or lyophilised) were mixed with dried KBr to make translucent IR pellets. All spectra were recorded at room temperature. To determine the percentage of water absorption, swelling studies were performed in phosphate-buffered saline (PBS, 0.1 M) at pH 7.4 and 37 °C [53,54]. The dry weight of the scaffold was noted (*Wo*). Scaffolds were then placed in PBS and taken out after 24 h. Surface adsorbed water was removed with filter paper and the wet weight was recorded (*Ww*). The swelling ratio was calculated as the weight ratio of the swollen versus dried sample, using the following formula:

Swelling ratio = (*Ww* − *Wo*)/*Wo* (1)

The thermal behaviour of the scaffolds was measured by thermogravimetric analysis (TGA) with a TGA Seiko Exstar 6300 instrument. The weight change experienced by the different scaffolds in relation to the thermal treatment applied was evaluated at a uniform heating rate of 10 °C/min to a maximum of 600 °C in a nitrogen atmosphere with a purge rate of 20 mL/min. About 10 mg of the sample was heated using Al_2_O_3_ crucibles. Scaffold conductivity was measured by using a four-point method [55]. Particularly, a constant current (1 mA) was applied between contact points made of silver electrodes by using a Fluke 8840 digital multimeter. Two of the contact points were placed at the edges of the scaffold cylinder in the same longitudinal plane (1 cm in length) with a pair of micromanipulators. The voltage drop in the scaffold surface was then measured by using another two silver electrodes located at the scaffold surface by slightly pressuring the scaffold with another pair of micromanipulators. The accuracy of the voltage measurement was approximately 1 μV. Conductivity was then calculated by using the following equation:

σ = [*L*/(π*r*^2^·*R*)] (2)

where *L* is the distance between the electrodes on the upper part of the scaffold, *R* is the measurement recorded by the multimeter and *r* the radius of the scaffold cylinder. The complex Young’s modulus of the samples was measured at 1 Hz in a triple-point bending configuration by using a DMA e7 dynamic mechanic analyser (Perkin-Elmer) [56]. The force used in the experiment was chosen so as not to produce strains larger than 0.3% in order to maintain the mechanical response within the linear range. Data were collected in triplicate. The error in the measurement was estimated to be around 20% (intrinsic to the measurement technique), which allowed comparisons between the outgoing data.

### 3.5. Biocompatibility Studies In Vitro

Prior to cell culture, scaffolds (ca. 4.5 mm in diameter, ca. 3 mm in thickness) were sterilized by UV radiation for 20 min per side and preconditioned in culture medium for 24 h to favour cell attachment. Cell culture studies were performed with human osteoblast-like Saos-2 cells. A total of 10^5^ cells were seeded on the top part of the scaffolds and allowed to attach for 5 min before adding cell culture media to submerge the entire samples. DMEM (Dulbecco’s Modified Eagle Medium) supplemented with fetal bovine serum (10%), streptomycin (100 UI mL^−1^), penicillin (100 UI mL^−1^), and l-glutamine (1 mM) was used as culture medium. Cultures were maintained at 37 °C in a sterile incubator under a CO_2_ (5%) atmosphere for up to seven days and culture media were replaced every other day. Tissue culture polystyrene (TCP) was used as a control surface. To test cell viability, cells cultures were analysed by using a Live/Dead^®^ Viability kit according to manufacturer’s instructions (Life Sciences, Farmingdale, NY, USA). This kit is based on the use of calcein, retained inside live cells, and converted into a strongly green-light-emitting compound after contact with intracellular esterases, and ethidium homodimer-1 (EthD-1), a DNA-intercalating agent that emits orange/red fluorescence when inserted into the DNA double helix of dead cells. After staining, samples were visualized by using a Leica SP5 confocal laser scanning microscope. The fluorescence of both probes was excited by an argon laser tuned to 488 nm. After excitation, emitted fluorescence was separated by using a triple dicroic filter 488/561/633 and measured at 505–570 nm for green fluorescence (calcein) and 630–750 nm for red fluorescence (EthD-1). Physical reflexion from the scaffolds was also recorded after excitation at 488 nm to visualize the scaffold structure and the relative cellular location.

## 4. Conclusions

In summary, this work demonstrates the ability of the ISISA technique for preparing monolithic scaffolds with finely tailored microchanneled structures for biological applications. The use of ice as a structure directing agent avoids the employment of further chemicals to template the final structure (e.g., surfactants, block copolymers, etc.) and emphasizes the green features of this soft-templating approach that, overall, will ultimately favour the intrinsic biocompatibility of the resulting materials. The engineered scaffolds were structured in form of well-aligned microchannels and exhibit good mechanical properties and high surface area/volume ratios. Preliminary biocompatibility in in vitro tests with human osteoblast-like cells also reveal the ability of these structures to support cell growth, with increasing viability values for those scaffolds prepared from SN-type MWCNTs and low CS contents.

## Figures and Tables

**Table 1:** Water-binding capacity of MWCNT/CS scaffolds.

MWCNTs (%) *x*	CS1SN*x*H	CS4SN*x*H	CS1LN*x*H	CS4LN*x*H
2.5	1633.8	983.4	1702.7	1049.4
4	1492.3	856.4	1594.8	920.4
6	1229.0	745.4	1379.5	800.9
8	990.6	697.8	1022.1	765.9
10	832.0	600.4	896.9	629.9
12	728.0	543.3	756.9	582.8
15	614.2	503.2	647.5	524.1
18	545.1	---	512.2	---
21	455.2	---	---	---

## Supplementary Materials

The following are available online at www.mdpi.com/1996-1944/10/4/355/s1. Ice as a Green-Structure-Directing Agent in the Synthesis of Macroporous MWCNTs and Chondroitin Sulphate Composites. Figure S1: Representative TEM images of pristine (a), LN (b) and SN (c) MWCNTs. Figure S2: XPS spectra of O1s core level of LN (A) and SN (B) MWCNTs. Figure S3: SEM images of cross-sectioned (perpendicular to the direction of freezing) CS1LN6 scaffolds. Figure S4: Representative SEM images of cross-sectioned MWCNT/CS scaffolds prepared with different content of CS and MWCNTs (LN or SN), and frozen at a dipping rate of 5.9 mm·min^−1^. Figure S5: TGA thermogram of pristine CS. Figure S6: Representative SEM images of cross-sectioned CS3LN6 scaffolds: (a,b) before cross-linking (CS3LN6), (c,d) after cross-linking (CS3LN6H) and (e,f) after swelling in PBS. Figure S7: TGA thermograms of CS1LN10 (a) and CS1LN10H (b) scaffolds. Figure S8: FT-IR spectra of CS in its original form and after cross-linking with HMDI vapors (CSH). Figure S9: Conductivity and Young’s modulus values of CSxLN6 and CSxLN10 scaffolds prepared with different concentration of CS, and either cross-linked (*c*) or non-cross-linked (*nc*). Figure S10: Conductivity and Young’s modulus values of CS1LN6 and CS1SN6 scaffolds prepared at different dipping rates, and either cross-linked (*c*) or non-cross-linked (*nc*). Table S1: Conductivity and Young’s modulus values of LN-composed MWCNT/CS scaffolds prepared with different concentrations of LN and CS, and either cross-linked (*c*) or non-cross-linked (*nc*). Table S2: Conductivity and Young’s modulus values of SN-composed MWCNT/CS scaffolds prepared with different concentrations of SN and CS, and either cross-linked (*c*) or non-cross-linked (*nc*). Table S3: Conductivity and Young’s modulus values of CS1LN6 and CS1SN6 scaffolds prepared at different dipping rates, and either cross-linked (*c*) or non-cross-linked (*nc*).
