# Supplementary material for: Ice as a Green-Structure-Directing Agent in the Synthesis of Macroporous MWCNTs and Chondroitin Sulphate Composites
Source: Materials (Basel). 2017 Mar 28;10(4):355. doi: 10.3390/ma10040355 (PMC5506963; doi:10.3390/ma10040355)
Supplement: Supplementary file 1 [file materials-10-00355-s001.pdf]

Supplementary Materials: Ice as a Green-Structure-Directing Agent in the Synthesis of Macroporous MWCNTs and Chondroitin Sulfate Composites

Stefania Nardecchia, MaríaConcepción Serrano, Sara García-Argüelles, Marcelo E. H. Maia Da Costa, María Luisa Ferrer and María C. Gutiérrez

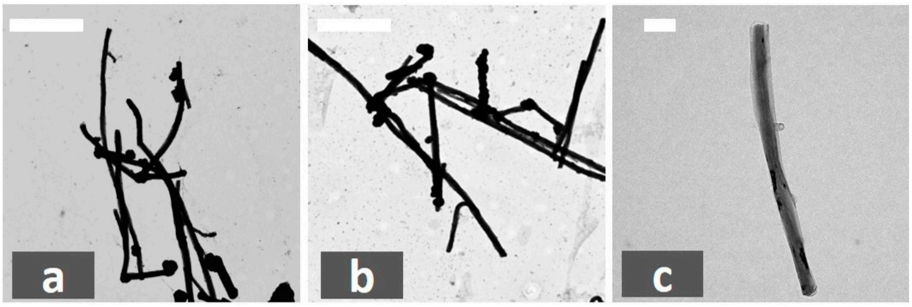

Figure S1. Representative TEM images of pristine (**a**), LN (**b**) and SN (**c**) MWCNTs. Scale bars represent 2 (**a**,**b**) and 0.2 (**c**) μm.

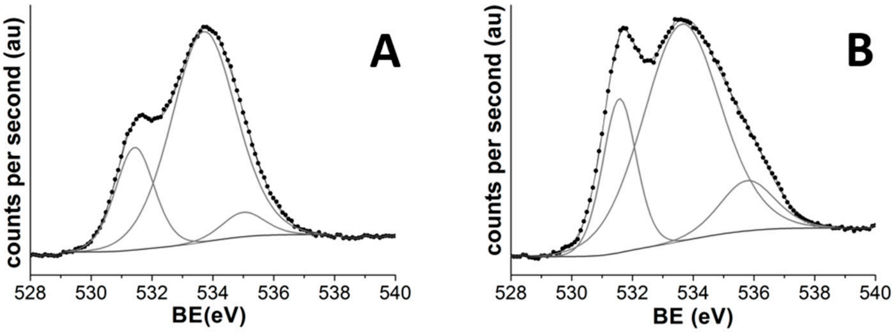

Figure S2. XPS spectra of O1s core level of LN (**A**) and SN (**B**) MWCNTs.

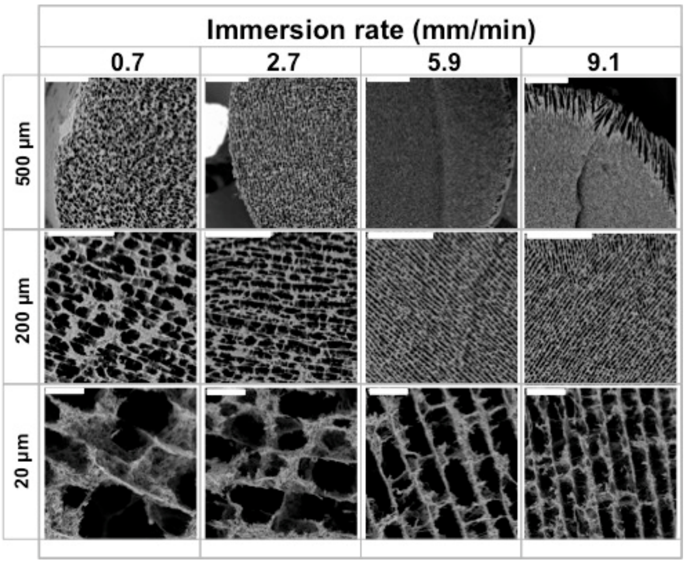

Figure S3. SEM images of cross-sectioned (perpendicular to the freezing direction) CS1LN6 scaffolds. The dipping rates were 0.7, 2.7, 5.9 and 9.1 mm·min^{-1}. Scale bars represent 500, 200 and 20 μm as indicated in the figure.

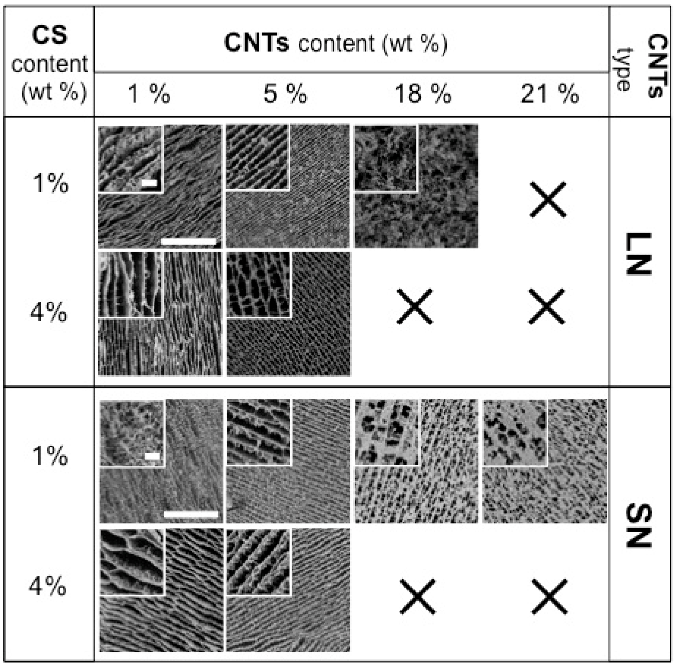

Figure S4. Representative SEM images of cross-sectioned MWCNT/CS scaffolds prepared with different content of CS and MWCNTs (either LN or SN), and frozen at a dipping rate of 5.9 mm·min^{-1}. Scale bars represent 200 µm and 20 µm in the inset images.

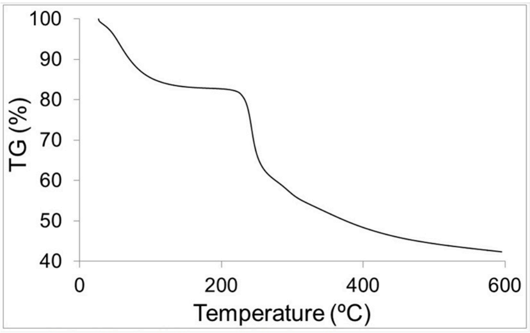

Figure S5. TGA thermogram of pristine CS.

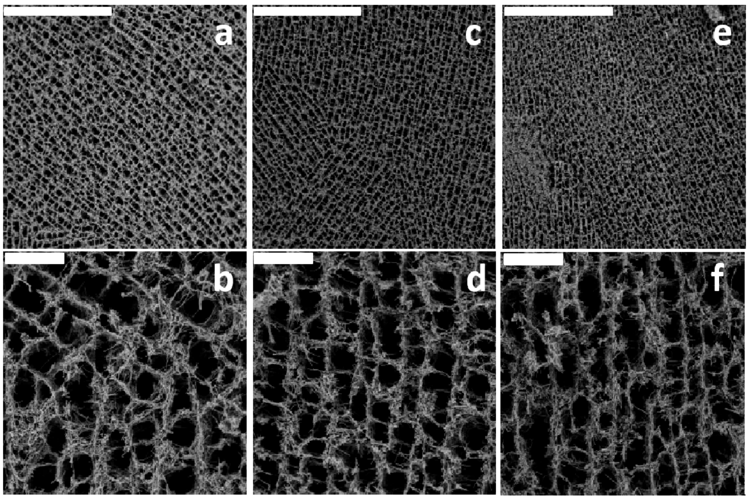

Figure S6. Representative SEM images of cross-sectioned CS3LN6 scaffolds: (**a**,**b**) before cross-linking, (**c**,**d**) after cross-linking and (**e**,**f**) after swelling in PBS. Scale bars represent 200 (**a**,**c**,**e**) and 20 (**b**,**d**,**f**) µm.

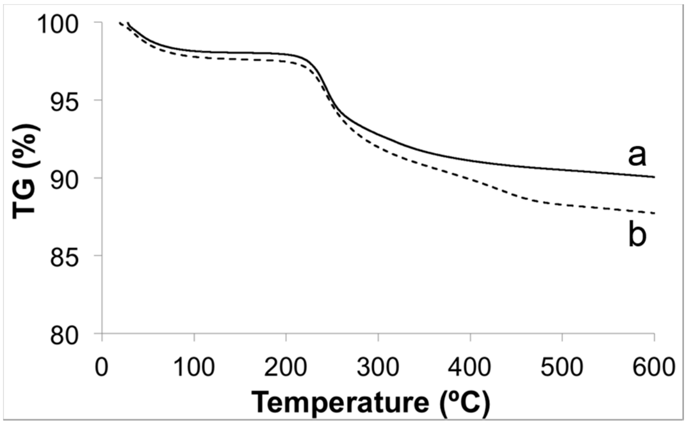

Figure S7. TGA thermograms of CS1LN10 (**a**) and CS1LN10H (**b**) scaffolds.

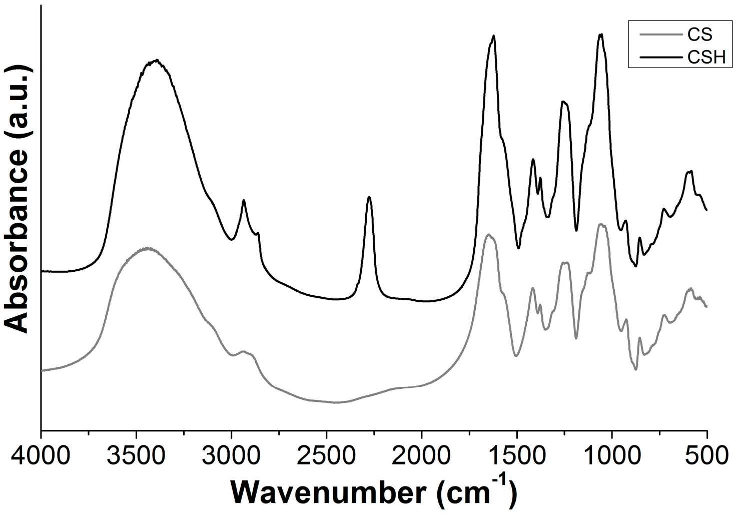

Figure S8. FT-IR spectra of CS in its original form and after cross-linking with HMDI vapors (CSH).

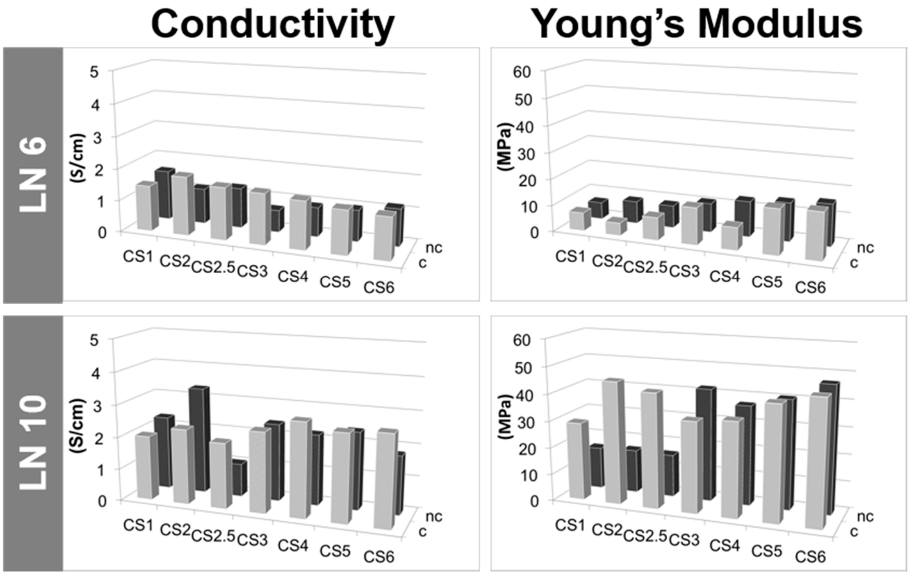

Figure S9. Conductivity and Young's modulus values of CSxLN6 and CSxLN10 scaffolds prepared with different concentration of CS, and either cross-linked (*c*) or non-cross-linked (*nc*).

Table S1. Conductivity and Young's modulus values of LN-composed MWCNT/CS scaffolds prepared with different concentrations of LN and CS, and either cross-linked (*c*) or non-cross-linked (*nc*).

**Scaffold**		**Conductivity (S/cm)**		**Young's modulus (MPa)**	
CS (%wt)	LN (%wt)	nc	c	nc	c
1	2.5	0.34	0.26	2.4	2.8
1	5	0.92	1.30	6.2	7.1
1	6	1.57	1.44	6.4	9.3
1	8	1.48	1.93	14.0	19.2
1	10	2.30	2.01	15.6	19.3
1	12	2.58	2.55	18.0	20.1
1	15	1.85	2.37	18.7	21.3
2	2.5	0.35	0.45	3.1	5.0
2	5	1.09	1.20	9.1	7.5
2	6	1.11	1.85	8.4	9.5
2	8	1.86	1.87	15.3	18.2
2	10	3.32	2.34	16.1	21.8
2	12	3.46	2.56	33.0	22.4
2	15	2.54	2.35	49.3	48.2
2.5	6	1.26	1.65	8.4	8.6
2.5	10	1.03	2.04	15.8	42.6
3	6	0.69	1.60	10.9	14.0
3	10	2.41	2.50	41.9	33.7
4	2.5	0.18	0.33	9.2	9.3
4	5	0.89	1.16	12.0	11.1
4	6	0.92	1.51	13.3	19.4
4	8	1.29	2.14	18.0	23.0
4	10	2.20	2.80	37.0	35.0
4	12	3.82	3.20	38.0	37.2
4	15	3.46	2.98	49.0	48.2
5	6	0.98	1.38	14.5	17.0
5	10	3.55	2.70	40.5	42.3
6	2.5	0.21	0.41	9.1	13.9
6	5	0.97	1.00	18.9	16.9
6	6	1.16	1.32	15.7	17.6
6	8	1.80	2.00	40.0	40.8
6	10	2.21	2.80	46.9	45.7
6	12	3.85	3.39	55.8	44.5
6	15	4.80	3.25	56.1	54.8

Table S2. Conductivity and Young's modulus values of SN-composed MWCNT/CS scaffolds prepared with different concentrations of SN and CS, and either cross-linked (*c*) or non-cross-linked (*nc*).

**Scaffold**		**Conductivity (S/cm)**		**Young's modulus (MPa)**	
CS (%wt)	SN (%wt)	nc	c	nc	c
1	2.5	---	---	1.9	2.7
1	5	0.11	0.13	4.6	5.8
1	6	0.23	0.20	3.7	5.3
1	8	0.33	0.25	10.9	12.3
1	10	0.51	0.49	14.4	22.5
1	12	0.71	0.77	21.8	27.0
1	15	1.33	1.25	33.7	27.8
4	2.5	---	---	3.2	3.8

4	5	0.02	0.06	5.1	10.9
4	6	0.36	0.13	11.4	11.7
4	8	0.36	0.49	13.2	13.2
4	10	0.35	0.66	29.2	29.3
4	12	0.64	0.68	31.2	30.5
4	15	0.60	0.73	41.1	42.5

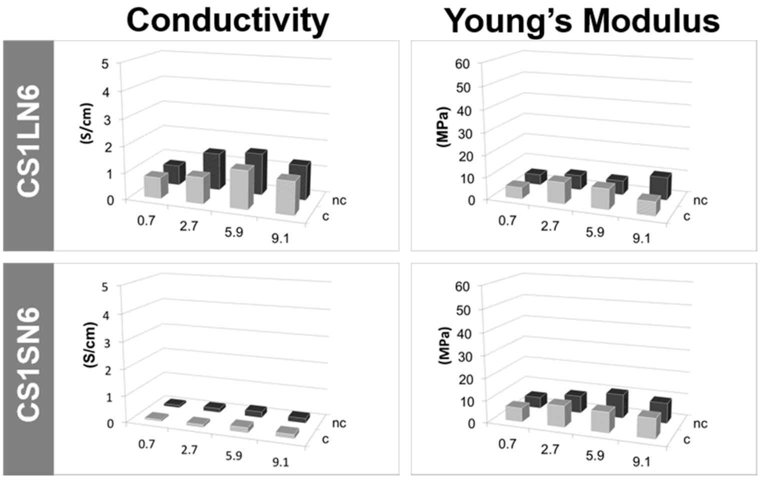

Figure S10. Conductivity and Young's modulus values of CS1LN6 and CS1SN6 scaffolds prepared at different dipping rates, and either cross-linked (*c*) or non-cross-linked (*nc*).

Table S3. Conductivity and Young's modulus values of CS1LN6 and CS1SN6 scaffolds prepared at different dipping rates, and either cross-linked (*c*) or non-cross-linked (*nc*).

**Scaffold**	**Dipping Rate**	**Conductivity (S/cm)**		**Young's modulus (MPa)**	
		nc	c	nc	c
CS1LN6	0.7	0.76	0.80	4.8	5.2
	2.7	1.40	1.00	6.6	9.8
	5.9	1.57	1.44	6.4	9.3
	9.1	1.30	1.25	10.2	6.3
CS1SN6	0.7	0.09	0.08	5.0	6.4
	2.7	0.15	0.10	7.8	9.7
	5.9	0.22	0.20	10.7	9.3
	9.1	0.17	0.17	9.1	9.1

© 2017 by the authors. Submitted for possible open access publication under the terms and conditions of the Creative Commons Attribution (CC-BY) license (http://creativecommons.org/licenses/by/4.0/).
